# IGF-II, IGFBP-4, -6, and -7, and HMGB1 show changes in follicular fluid in PCOS

**DOI:** 10.3389/fendo.2026.1856759

**Published:** 2026-06-05

**Authors:** Veronica Buia, Cecilia Catellani, Stefania Croci, Beatrice Righi, Daria Morini, Angela Immacolata Falbo, Alessia Nicoli, Alessandro De Fanti, Lucia Belloni, Lorenzo Aguzzoli, Maria Teresa Villani, Chiara Sartori, Maria Elisabeth Street

**Affiliations:** 1 Unit of Clinical Immunology, Allergy and Advanced Biotechnologies, Azienda Unità Sanitaria Locale - IRCCS di Reggio Emilia, Reggio Emilia, Italy; 2 Unit of Pediatrics, Department of Mother and Child, Azienda Unità Sanitaria Locale - IRCCS di Reggio Emilia, Reggio Emilia, Italy; 3 Fertility Center, Department of Mother and Child, Azienda Unità Sanitaria Locale - IRCCS di Reggio Emilia, Reggio Emilia, Italy; 4 Unit of Paediatrics, University Hospital of Parma, Parma, Italy; 5 Department of Medicine and Surgery, University of Parma, Parma, Italy

**Keywords:** follicular fluid, HMGB1, IGF, IGFBP, PCOS

## Abstract

**Introduction:**

Polycystic ovary syndrome (PCOS) is a multisystem endocrine and metabolic disease characterized by chronic low-grade inflammation, ovulatory dysfunction, hyperandrogenism, and insulin resistance. Chronic inflammation modifies the IGF system that regulates ovarian function and glucose metabolism. HMGB1 is an alarmin related with both inflammation and insulin sensitivity, and we previously described the increased follicular fluid concentration of HMGB1 in PCOS. This study aimed to investigate changes in IGF system protein concentrations in follicular fluid from PCOS with respect to control subjects.

**Methods:**

A total of 70 PCOS diagnosed according to the Rotterdam criteria and 70 control women were enrolled. Subjects were stratified based on BMI<25 (underweight/normal weight) or ≥25 (overweight/obese). PCOS and controls underwent induction of follicular development for IVF, and follicular fluid was collected during oocyte retrieval. IGF-I, IGF-II, IGFBP-1-7, and HMGB1 concentrations were measured in follicular fluids using specific ELISA kits.

**Results:**

IGF-II and IGFBP-4 were lower whereas IGFBP-6 and IGFBP-7 were increased in PCOS compared with controls. Instead, IGF-I and IGFBP-1, -2, and -3 were similar. IGFBP-5 was undetectable. HMGB1 was increased in PCOS. Stratification based on BMI showed that under/normal weight, PCOS women had lower IGF-II and IGFBP-4 and increased IGFBP-7 in follicular fluids compared with controls. IGFBP-2 was higher in under/normal weight controls with respect to their overweight/obese counterparts. When considering the molar ratios, IGF-II bioavailability was significantly lower in PCOS whereas IGF-I bioavailability was unchanged. IGFBP-3 correlated with IGFBP-4 in controls, and IGFBP-2 correlated with IGFBP-1 in PCOS. HMGB1 correlated with IGFBP-2 in PCOS and with IGF-I in controls.

**Conclusion:**

The reduction in IGF-II and IGFBP-4 in FF in PCOS is compatible with reduced follicular development. The changes in IGF-II, IGFBP-4, and IGFBP-7 in the under/normal weight PCOS women suggests that these changes are specific to PCOS. The correlation between HMGB1 and IGFBP2 is compatible with the increased inflammatory status in PCOS. The increase of IGFBP-6 and -7 is a novel finding that opens the way for further research.

## 1 Introduction

Polycystic ovary syndrome (PCOS) is a multisystem endocrine and metabolic disorder which is reported to be the most common endocrine disease in women of reproductive age (1, 2), affecting 5%-18% depending on the criteria used for diagnosis (3).

Its etiology is still unclear; however, genetic predisposition, epigenetic factors, gestational environment, and lifestyle play an important role (4). PCOS typically presents during adolescence and is more frequent in overweight or obese women (5).

In adults, the diagnosis of PCOS is based on the presence of at least two of the following characteristics: (I) clinical/biochemical signs of hyperandrogenism, (II) ovulatory dysfunction, and (III) polycystic ovarian morphology on ultrasound or elevated anti-mullerian hormone (AMH) levels, after the exclusion of other causes (6). Moreover, PCOS is associated with systemic low-grade inflammation and often with insulin-resistance (70%-75% of PCOS women) (7) which contribute to PCOS comorbidities such as infertility, hyperinsulinemia, impaired glucose tolerance, type 2 diabetes, dyslipidemia, obesity, and increased cardiovascular risk (8–11).

In PCOS, an increase in circulating pro-inflammatory cytokines has been reported to contribute to ovarian dysfunction (12–17) and to hamper insulin sensitivity (18, 19). We previously described increased high-mobility group box 1 (HMGB1) content in follicular fluid (FF) of PCOS patients with respect to healthy control women (20). HMGB1 is involved in inflammatory diseases (21) and different insulin resistance/hyperinsulinemia-related disorders such as obesity and type 2 diabetes mellitus (22). HMGB1 is linked also to glucose metabolism, a sensitive pathway in PCOS (23).

The insulin-like growth factor (IGF) system is a complex network of proteins comprising insulin, IGF-I, IGF-II, and IGF-binding proteins (IGFBP1-7), the latter being involved in the regulation of bio-availability and activity of IGFs and IGFBP-2, -4, and -5 having mainly an inhibitory action on IGFs (24–26). The IGF system is involved in the regulation of glucose metabolism (27) and is altered in chronic inflammation where both circulating IGF-I and -II can be reduced (25, 28). An interesting study showed that both low and high IGF-I levels can be associated with insulin resistance (29). IGFBP-1 and IGFBP-2 are negatively regulated by insulin and glucose. Moreover, IGFBP-2 has been reported to increase under conditions of increased/chronic inflammation (25, 30–32). The IGF system has a relevant role also in obesity (33). Indeed, IGF-1 circulating levels have been described to be low in obese subjects, particularly in those with increased adiposity, inflammation, dyslipidemia, and hyperuricemia (34). Moreover, low serum levels of IGF-II and IGFBP-2 have been reported in both obese children and adults (31, 35, 36), and lower serum IGFBP-1 levels have been associated with an increased risk of metabolic syndrome (37).

The IGF system is of importance also for the regulation of ovarian function (38). Both IGF-I and IGF-II promote follicular maturation in mammals (39–41), and their effects depend on follicular development stage and on the regulation by IGFBPs (42). IGF-I and IGF-II levels have been described to be reduced in PCOS ovaries (43). Conversely, free IGF-I circulating levels have been described to be increased and to correlate negatively with the total follicle number and with IGFBP-1 levels (44). IGFBP-1 has been found to play different roles in the female reproductive tract; in the ovary it would impact ovulation, follicular growth and steroidogenesis (45). IGFBP-2 was reported to be higher in PCOS and atretic follicles from normally cycling women compared with normal healthy estrogenic follicles suggesting a role in normal folliculogenesis (46). IGFBP-3 levels would be of importance for oocyte maturation and embryo development (41). Both IGFBP-4 and -5 would contribute to follicle development. In normal physiology, intact IGFBP-4 has been observed to increase in small antral follicles and to be lower in preovulatory follicles, whereas IGFBP-5 would show an opposite trend (40). There are no data to our knowledge on intrafollicular IGFBP-6 levels in humans. However, IGFBP-6 expression levels were reported to be increased in bovine theca cells in the final stages of follicular maturation (47); conversely, in cycling ewes, they were decreased in large atretic follicles compared with normal follicles (48). Recently, a bulk RNA-seq analysis of PCOS granulosa cells evidenced that IGFBP-7 mRNA levels were increased in granulosa cells from patients with PCOS and the knockdown of the IGFBP-7 gene in mice with PCOS led to decreased ovarian cell apoptosis, increased proliferation, and androgen synthesis (49). A recent proteomic analysis evidenced that the IGF system was among the most enriched pathways in FF in PCOS (50), suggesting that it is worth to be investigated to better understand this complex condition.

The aim of this study was to investigate changes in the IGF system in FF from PCOS women compared with controls and to evaluate any relationships with body mass index (BMI) and HMGB1 as a marker of both inflammation and altered glucose metabolism. In particular, we hypothesized that the content and bioavailability of some IGF system proteins could change in PCOS and that these changes could be dependent on BMI.

## 2 Materials and methods

### 2.1 Subjects

At the time of oocyte retrieval for *in vitro* fertilization (IVF) procedures, 70 patients with PCOS [CA (years):34.1 ± 4.7 years; BMI (kg/m^2^):25.6 ± 5.6; hirsute (n):16; amenorrhoic (n):6; oligomenorrhoic (n):31; regularly cycling (n):33] and 70 regularly cycling women (control subjects) [CA (years):36.8 ± 3.8 years; BMI (kg/m^2^):24.0 ± 5,0; none hirsute; all regularly cycling (n):70] were enrolled in the study by the Fertility Center, Department of Mother and Child, Arcispedale Santa Maria Nuova, AUSL - IRCCS of Reggio Emilia. The main clinical features and findings at ultrasound are reported in **Supplementary Table 1**.

PCOS patients were diagnosed according to the Rotterdam criteria (51). The presence of at least two symptoms among oligo/amenorrhea, clinical or biochemical signs of hyperandrogenism, and polycystic ovarian morphology at ultrasound were considered, and additional criteria whenever possible (6). Control subjects were women undergoing IVF because of tubal or idiopathic infertility causes, with normal endocrine exams and regular menstrual cycles. Control subjects were screened to exclude ovulatory dysfunctions or mild forms of PCOS. Exclusion criteria were the presence of tumors, endometriosis, celiac disease, genetic or chronic diseases, Cushing syndrome, hyperprolactinemia, and dimorphisms. All participants did not receive any additional treatment for at least 2 months before IVF except for the ovarian stimulation therapy.

### 2.2 Hormonal stimulation

All subjects enrolled in this study underwent the same stimulation protocol. In detail, ovarian hormonal stimulation was performed according to a long luteal gonadotropin-releasing hormone (GnRH) agonist depot protocol. After the confirmation of complete ovarian downregulation by means of biochemical and instrumental analyses, recombinant FSH (rFSH) was administered in the first 5 days using a starting dose tailored according to the patient’s age and antral follicle count. From day 6 of ovarian stimulation, the dose was modified according to the ovarian response. In case of appearance of dominant follicles, ovulation was stimulated by the injection of 10.000 IU hCG in the 24 h after the last injection of rFSH. Then, 36 h after hCG administration, oocyte retrieval was performed by ultrasonography-guided transvaginal aspiration. Estradiol (E2) serum concentrations at oocyte retrieval are reported in **Supplementary Table 1**.

### 2.3 Collection of FF samples

FF was aspirated from follicles with a diameter of 14–22 mm during oocyte retrieval and was processed immediately. FF was centrifuged for 10 min at 1,500×g, and the supernatant was transferred into fresh tubes and further centrifuged for 10 min at 2,000×g at room temperature for complete removal of red blood cells or debris. Samples were aliquoted and stored at −80 °C until assayed.

### 2.4 Biochemical assays

The following analytes were quantified in FF using specific ELISA kits: IGF-I (DG100B, R&D, Minneapolis, USA), IGF-II (DG200, R&D Minneapolis, USA), IGFBP-1 (EHIGFBP1, Invitrogen, Waltham, USA), IGFBP-2 (E05, Mediagnost, Reutlingen, Germany), IGFBP-3 (DGB300, R&D Minneapolis, USA), IGFBP-4 (EHIGFBP4, Invitrogen, Waltham, USA), IGFBP-5 (EHIGFBP5, Invitrogen, Waltham, USA), IGFBP-6 (EHIGFBP6, Invitrogen, Waltham, USA), IGFBP-7 (EH252RB, Invitrogen, Waltham, USA), HMGB1 (HMGB1 ELISA, IBL, Hamburg, Germany). Technical characteristics of the assays are reported in **Supplementary Table 2**. 17β-Estradiol (E2) serum concentrations were assayed the day of oocyte retrieval by means of the ADVIA Centaur Enhanced Estradiol Assay (IVD, Siemens AG, Munich Germany). IGFBP-5 in follicular fluid was always below the limit of detection of the kit and was not considered further (sensitivity: 8 ng/ml). IGF-I, IGF-II, IGFBP-1, IGFBP-2, IGFBP-3, IGFBP-4, IGFBP-6, and IGFBP-7 concentrations were considered also as nmol/L (nM) by multiplying the single values by 0.131, 0.134, 0.033, 0.035, 0.035, 0.038, 0.034, and 0.034, respectively (25, 52). Subsequently, the molar ratios of the single IGFs to each IGFBP were calculated to obtain an estimate of bioactive IGF-I and IGF-II in follicular fluid.

### 2.5 Statistical analysis

Statistical analysis was performed using the statistical package GraphPad Prism 10 (GraphPad Software, Boston, Massachusetts USA). Patients with PCOS and controls were stratified based on BMI <25 kg/m^2^ (PCOS: 17.6-24.9 kg/m^2^, n=37; controls: 17.2-24.9 kg/m^2^, n=49) (under/normal weight) or ≥25 kg/m^2^ (PCOS: 25.0-39.3 kg/m^2^, n=32; controls: 25.0-38.7 kg/m^2^, n=20) (overweight/obese) in order to evaluate differences in IGF and IGFBP content in FF related with BMI. Unpaired Student’s T-test was used to evaluate the differences between PCOS and control groups. One-way ANOVA analysis was performed to study the differences among the following subgroups: underweight/normal weight (BMI<25) control subjects, overweight/obese (BMI≥25) control subjects, underweight/normal weight (BMI<25) PCOS, overweight/obese (BMI≥25) PCOS. Šídák’s multiple comparisons test was used to assess differences between pairs of group means. Pearson’s correlation analysis was performed to investigate the correlation between the analyzed parameters. Only significant correlations are reported.

### 2.6 Ethical approval

The study was approved by the Ethical Committee of Reggio Emilia (project ID: PCOS2_15_17). Written informed consent was obtained from all subjects as appropriate.

## 3 Results

### 3.1 IGF system protein levels and HMGB-1 in FF in PCOS with respect to controls

**Figure 1:**
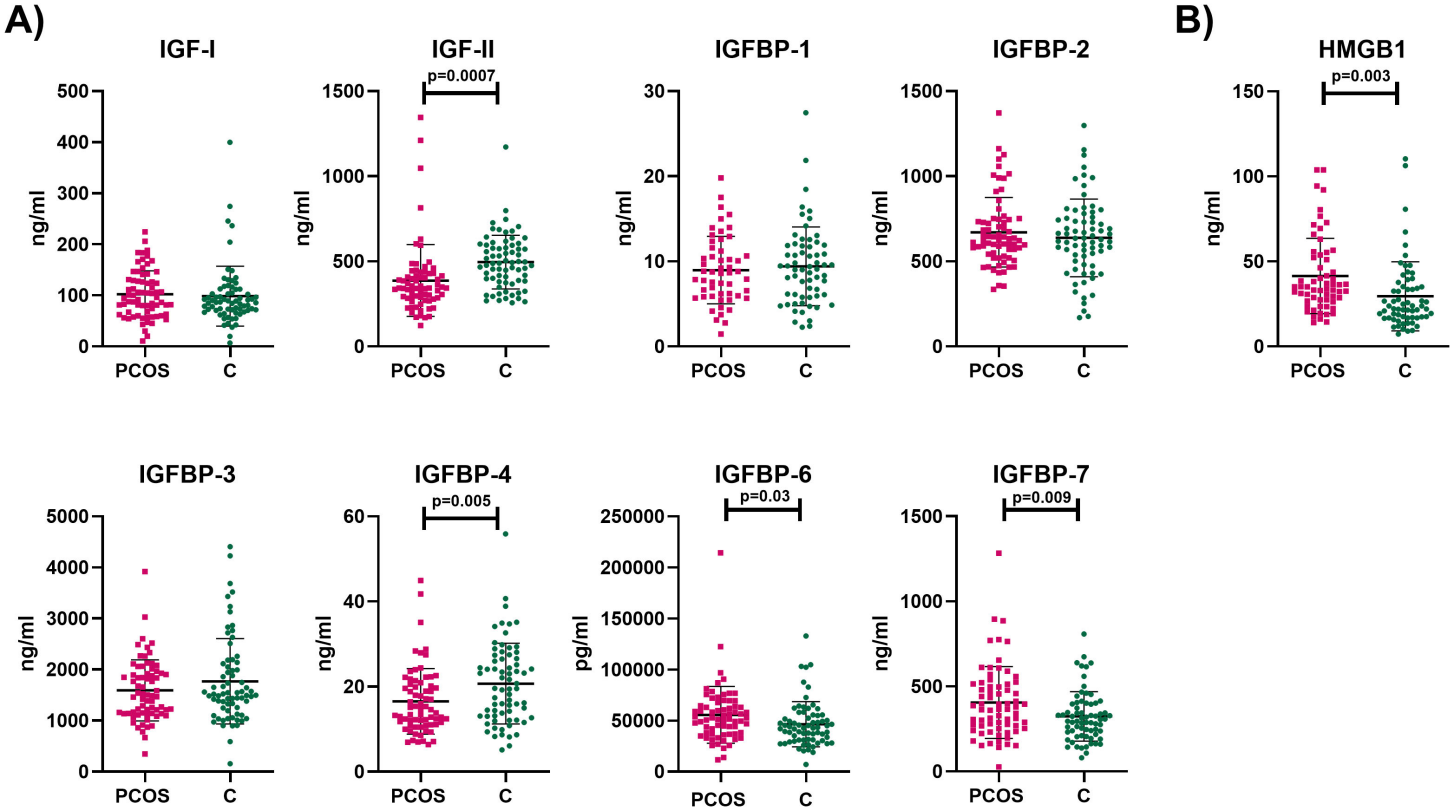
IGFs, IGFBPs, and HMGB1 levels in FF of women with PCOS and controls. **(A)** IGF system protein levels in FF of women with PCOS compared with controls PCOS n=70; controls (C) n=70. **(B)** HMGB1 levels in FF of women with PCOS compared with controls. Data are reported as mean ± SD. The differences between the two groups were analyzed using unpaired Student’s T-test, and p-values< 0.05 were considered significant.

IGF system peptide and HMGB-1 levels in FF from PCOS and control women are reported in **Figure 1A**. IGF-I (102.1 ± 46.1 ng/ml vs. 98.5 ± 58.7 ng/ml, n.s.), IGFBP-1 (9.0 ± 3.4 ng/ml vs. 9.4 ± 4.6 ng/ml, n.s.), IGFBP-2 (671.2 ± 204.7 ng/ml vs. 638.0 ± 228.0 ng/ml, n.s.), and IGFBP-3 (1,592.0 ± 597.3 ng/ml vs. 1,770.0 ± 835.6 ng/ml, n.s.) levels were similar in PCOS and control subjects. IGFBP-5 was undetectable in most FF samples. IGF-II (387.2 ± 210.5 ng/ml vs. 495.7 ± 157.9 ng/ml; p=0.0007) and IGFBP-4 (16.5 ± 7.7 ng/ml vs. 20.7 ± 9.5 ng/ml; p=0.005) were lower in FF from PCOS women compared with controls, whereas IGFBP-6 (55,570 ± 27,793 pg/ml vs. 46,417 ± 22,110 pg/ml; p=0.03) and IGFBP-7 (405.9 ng/ml ± 211.6 ng/ml vs. 324.1 ng/ml ± 145.8 ng/ml; p=0.009) were higher.

HMGB1 levels in FF were higher in PCOS compared with controls (41.5 ± 22.1 ng/ml vs. 29.5 ± 20.4 ng/ml; p=0.003) (**Figure 1B**).

#### 3.1.1 Impact of different phenotypes in the PCOS group

In order to clarify whether pooling the different PCOS phenotypes in the PCOS group may obscure or dilute phenotype-specific effects, we performed a phenotype-stratified subanalysis for IGF-II, IGFBP-4, IGFBP-6, and IGFBP-7 considering the following comparisons: hirsute vs. not hirsute PCOS and oligo/amenorrhoeic PCOS vs. regularly cycling PCOS. As reported in **Supplementary Figure 1**, we did not find any differences among groups.

### 3.2 IGF/IGFBP molar ratios in FF

**Figure 2:**
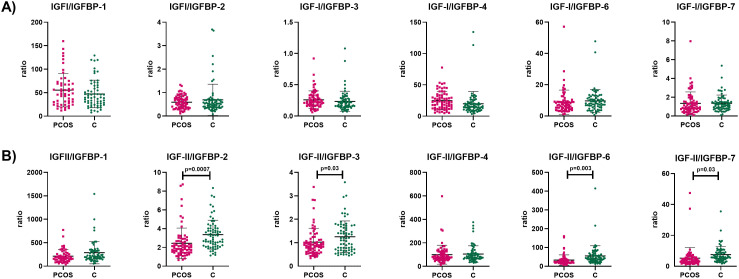
IGF to IGFBP molar ratios in FF from women with PCOS with respect to controls. **(A)** IGF-I and **(B)** IGF-II to IGFBP molar ratios in FF from PCOS n=70; controls (C) n=70. Data are reported as mean ± SD. p-values <0.05 were considered significant.

The molar ratios of IGFs to IGFBPs in both PCOS and controls are reported in **Figure 2**. No differences were found for IGF-I molar ratios between PCOS and controls (**Figure 2A**), whereas IGF-II/IGFBP-2 (p=0.0007), IGF-II/IGFBP-3 (p=0.03), IGF-II/IGFBP-6 (p=0.003), and IGF-II/IGFBP-7 (p=0.03) molar ratios were significantly lower in the PCOS group with respect to controls (**Figure 2B**).

### 3.3 IGF system and HMGB1 levels in FF in PCOS with respect to controls according to BMI

**Figure 3:**
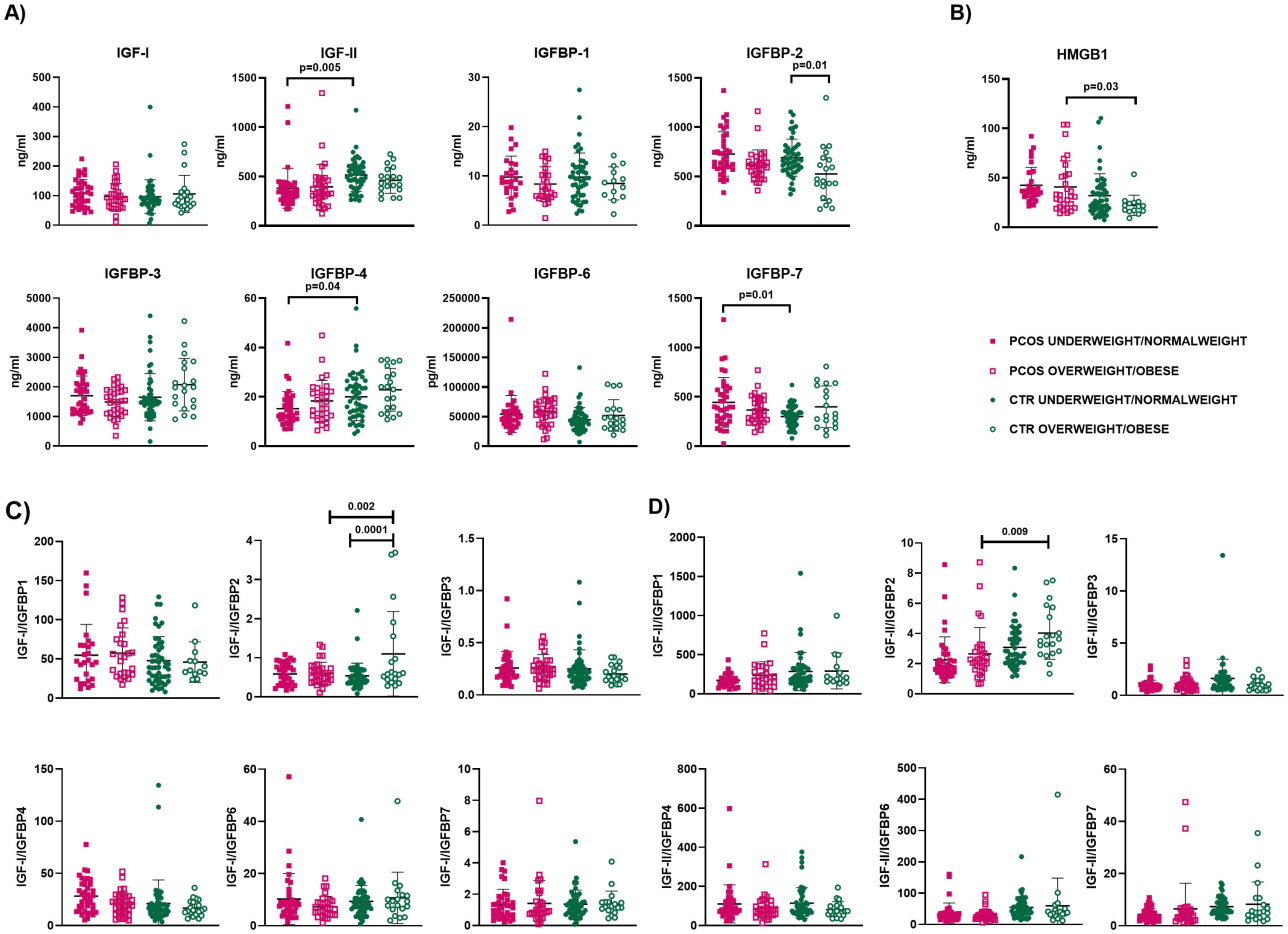
IGF system and HMGB1 protein levels in FF of women with PCOS compared with controls according to BMI **(A)** IGF system protein levels in FF of women with PCOS compared with controls according to BMI: underweight/normal weight PCOS (n=37), overweight/obese PCOS (n=32), overweight/normal weight C (n=49), and overweight/obese C (n=20). **(B)** HMGB1 levels in FF of women with PCOS compared with controls stratified based on BMI: underweight/normal weight PCOS (n=32), overweight/obese PCOS (n=29), overweight/normal weight C (n=49), and overweight/obese C (n=15). **(C)** IGF-I and **(D)** IGF-II to IGFBPs molar ratios in FF from women with PCOS with respect to controls according to BMI: underweight/normal weight PCOS (n=37), overweight/obese PCOS (n=32), overweight/normal weight C (n=49), and overweight/obese C (n=20). Data are reported as mean ± SD. p-values <0.05 were considered significant.

In under/normal weight women, IGF-II (380.7 ± 201.8 ng/ml vs. 514.1 ± 165.3 ng/ml; p=0.005) and IGFBP-4 (15.2 ± 6.9 ng/ml vs. 20.0 ± 9.7 ng/ml; p=0.04) FF levels were lower in PCOS women with respect to controls, whereas IGFBP-7 levels (444.7 ± 251.5 ng/ml vs. 300.6 ± 103.6 ng/ml; p=0.01) were higher (**Figure 3A**).

In overweight/obese women, IGFPB-2 FF levels were lower in controls with respect to their under/normal weight counterpart (525.2 ± 271.3 ng/ml vs. 690.9 ± 187.2 ng/ml; p=0.01) (**Figure 3A**). No differences in IGF-I, IGFBP-1, IGFBP-3, and IGFBP-6 levels were observed in PCOS versus controls according to BMI.

HMGB1 FF levels were increased in the overweight/obese PCOS women compared with the overweight/obese controls (40.7 ± 26.3 ng/ml vs. 22.1 ± 10.4 ng/ml; p=0.03, **Figure 3B**).

The IGF-I/IGFBP-2 molar ratio was increased in the overweight/obese controls compared with under/normal weight control women (p=0.0001) but was also significantly increased compared with the overweight/obese PCOS women (p=0.002) (**Figure 3C**).

The IGF-II/IGFBP-2 molar ratio was decreased in the overweight/obese PCOS women compared with the overweight/obese control women (p=0.009) (**Figure 3D**).

### 3.4 Correlation analyses

#### 3.4.1 BMI

**Figure 4:**
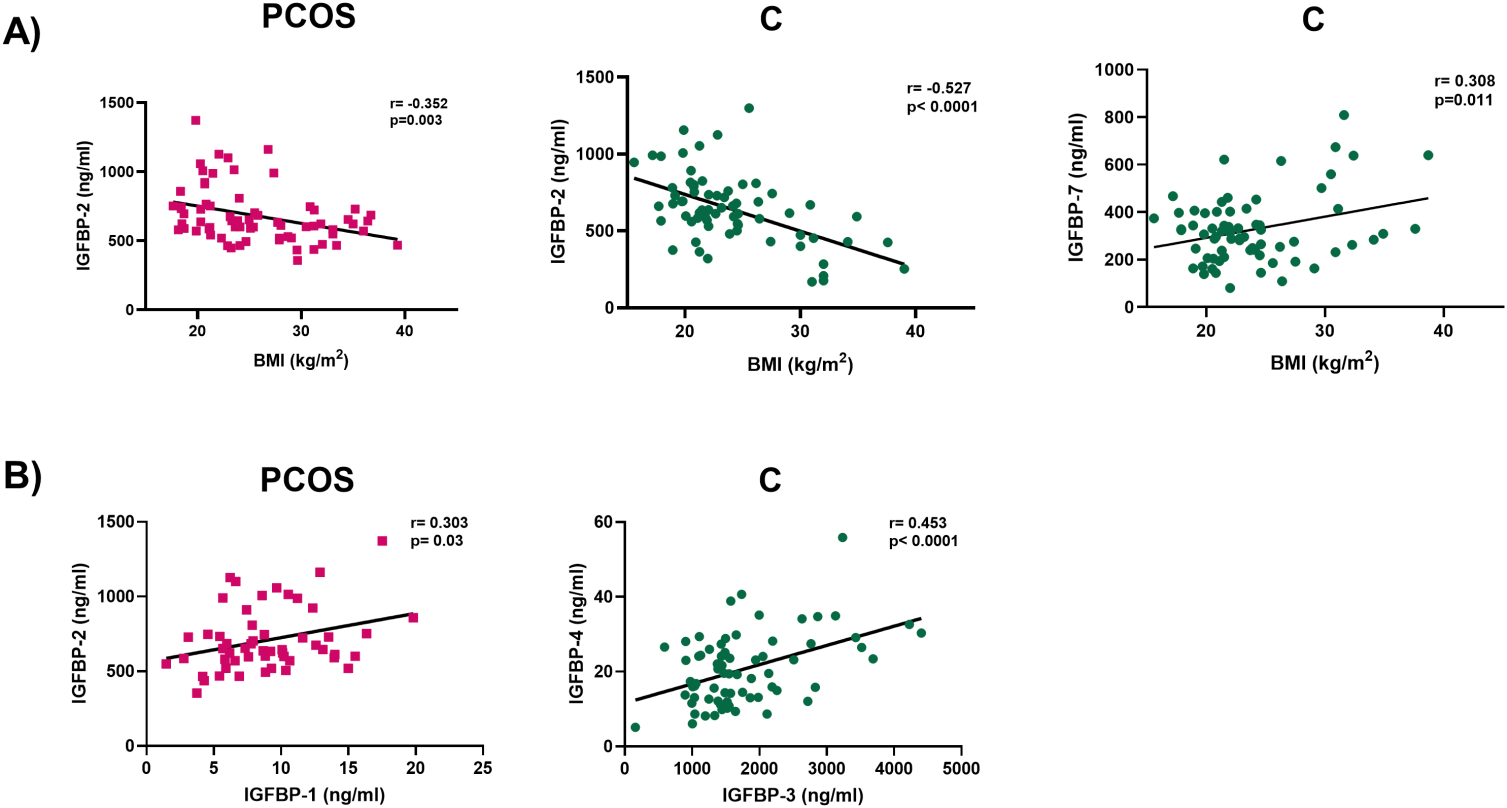
Correlations among IGF system peptides and BMI in FF in PCOS and control women. **(A)** Significant correlations among IGF system peptides and BMI in PCOS (n=70) and controls (C; n=70). **(B)** Correlations among IGF system peptides in PCOS (n=70) and controls (C; n=70). Only correlations which reached statistical significance (p<0.05) and with an R > 0.30 are shown.

The significant correlations between IGF system peptides in FF and BMI are reported in **Figure 4A**.

In PCOS, BMI was negatively associated with IGFBP-2 (r= −0.352, p=0.003). In controls, BMI was associated with IGFBP-2 (r= −0.527, p<0.0001) and IGFBP-7 (r= 0.308, p=0.011). Moreover, BMI was negatively associated with IGFBP-2 in both under/normal weight controls (r=−0.332, p=0.02*)* and overweight/obese controls (r=−0.620, p=0.004).

#### 3.4.2 IGF system peptides

Correlations were performed separately in controls and PCOS subjects. Significant correlations are reported for both groups in **Figure 4B**.

In PCOS women, IGFBP-1 correlated with IGFBP-2 (r=0.303, p=0.03) in FF. In control subjects, IGFBP-3 correlated with IGFBP-4 (r=0.453, p<0.0001).

#### 3.4.3 HMGB1

In women with PCOS, HMGB1 was positively associated with IGFBP-2 (r=0.341, p=0.007) whereas in controls, HMGB1 correlated with IGF-I (r= 0.303; p=0.02) (**Supplementary Figure 2**).

## 4 Discussion

The findings of this study showed changes in IGF system peptide content in FF from PCOS women and confirmed higher levels of HMGB1 in PCOS. Specifically, we observed a reduction in IGF-II and IGFBP-4 and an increase in IGFBP-6 and IGFBP-7. Overall IGF-II bioavailability was found to be reduced in FF in PCOS.

When considering BMI, under/normal-weight PCOS women had lower IGF-II, IGFBP-2, and IGFBP-4 contents and increased IGFBP-7 in FF compared with controls, whereas in obese PCOS women, such changes were not so evident suggesting that PCOS is associated with changes that are further modified by obesity. This is consistent with increased IGF-I and IGF-II to IGFBP-2 molar ratios in FF being observed in obese healthy women, but a further reduction of these ratios was observed in obese PCOS women. Furthermore, BMI was negatively associated with IGFBP-2 content in FF in both PCOS and controls. In these latter, BMI was positively associated with IGFBP-7 FF content. In control women, IGFBP-3 and IGFBP-4 FF contents were associated, whereas in PCOS, IGFBP-1 and IGFBP-2 were the peptides to be correlated.

HMGB1 was confirmed to be increased in FF in PCOS and obesity increased further its levels and was associated with IGFBP-2 FF content. In controls, HMGB-1 was found to be negatively and weakly associated with IGF-I FF content.

The finding of a reduction in IGF-II levels in FF from PCOS patients is consistent with few previous findings (44). As these changes were observed in the under/normal-weight PCOS subjects, this supports the hypothesis that these changes are specific to PCOS. The lower IGF-II bioavailability in PCOS, confirmed by the molar ratios of IGF-II to IGFBPs, in consideration of the reported functions of IGF-II in the ovary, would suggest a negative effect on follicular development that is a distinctive feature of PCOS (39, 40, 42, 53). IGF-II bioavailability was reduced in the FF from obese PCOS women compared with controls, but we did not find a reduced IGF-I content at variance with Barreca et al. (43) possibly due to the fact that these authors used a different detection method (RIA), and the BMI of the patients was not taken into account. IGF-I mRNA levels were previously reported to be reduced in bovine cystic follicles (54); however, differences in gene expression do not always reflect changes in protein levels, and findings in bovines may not reflect exactly findings in humans.

We did not find any differences in IGFBP-1 levels between PCOS and controls. The data available to date refer to blood concentrations, where a decrease in IGFBP-1 levels has been reported in insulin-resistant PCOS women (55), and insulin and IGFBP-1 serum concentrations are well known to be negatively associated (56). Serum probably cannot be considered as a proxy of follicular fluid, as previously supported by other studies; these previous findings were likely related to body metabolic changes (57), with an increased risk of metabolic syndrome being a well-known comorbidity in women with PCOS (37).

Although IGFBP-2 levels in FF were not significantly different between the PCOS and control groups, we observed some differences, in particular relative to the IGF-I and IGF-II to IGFBP-2 molar ratios as these were lower in the obese PCOS women compared with the obese control women, with BMI being associated with IGFBP-2 in both groups, and with IGFBP-2 being associated with IGFBP-1 in FF in PCOS women. Taken together, these data suggest that metabolic changes associated with an increased BMI have a stronger effect than PCOS *per se*. As previously mentioned, IGFBP-2 serum concentrations have been reported to be reduced in obesity (31, 34–36), fatty liver disease, and type 2 diabetes mellitus (58, 59) that represent all possible comorbidities of PCOS, and all are associated with reduced insulin sensitivity.

In our cohort, IGFBP-3 follicular fluid levels were similar in PCOS and control women and were independent of BMI. These data are in line with a previous study in serum from PCOS women which reported similar serum IGFBP-3 levels in PCOS and controls (60) but at variance with one proteomic study reporting lower serum IGFPB-3 levels in PCOS compared with controls (61). Moreover, our findings are different from previous findings reporting reduced IGFBP-3 in PCOS FF (62).

IGFBP-4 levels were lower in PCOS with respect to controls and particularly in under/normal-weight PCOS women compared with under/normal-weight controls, suggesting that these changes are specific to PCOS. IGFBP-4 is of special interest as it has been reported to be involved with follicle development; particularly, intact IGFBP-4 FF levels have been found to be higher in small antral follicles and to decrease in preovulatory follicles in patients with ovarian cancer and no endocrine abnormalities (40). Our findings reflect the perturbation of PCOS ovulatory function characterized by reduced follicular development and is in line with the findings of a previous report on serum IGFBP-4 levels in PCOS (61).

IGFBP-5 was undetectable in most of our samples, possibly depending on the detection method used, and not allowing for any conclusion on possible roles related with ovarian function. We cannot exclude a matrix effect either. IGFBP-5 has been poorly investigated in human FF. To the best of our knowledge, only one paper described the IGF system including IGFBP-5 gene expression in follicles at different developmental stages, reporting changes from preovulatory follicles to small antral follicles with an opposite direction compared with IGFBP-4 (40). However, gene expression does not reflect necessarily protein content, and this could account for the extremely low amounts detected.

The increased IGFBP-6 levels in FF in PCOS is a novel finding. IGFBP-6 expression levels have been found to be increased in bovine theca cells in the final stages of follicular maturation (47), and to be decreased in large atretic follicles in cycling ewes (48), suggesting that IGFBP-6 is involved in the regulation of follicular maturation. This suggests that IGFBP-6 should be further studied in human ovarian function and in PCOS.

IGFBP-7 content in FF was also increased in PCOS compared with control subjects, in particular in under/normal-weight women, suggesting that these changes are likely specific to PCOS. This would be confirmed by a recent bulk RNA-seq analysis of PCOS granulosa cells where IGFBP-7 mRNA levels were found to be increased, and the knockdown of the IGFBP-7 gene, in a PCOS mouse model, led to increased granulosa cell proliferation, reduced apoptosis, and decreased testosterone secretion correcting PCOS-like features (49). This underscores the importance of IGFBP-7 in human ovarian function too and the need for further research. Finally, the weak positive association of BMI with IGFBP-7 FF content supports a worse outcome in obese PCOS women compared with the under/normal weight.

Finally, HMGB1 FF levels were increased in PCOS patients with respect to control subjects confirming previous findings obtained also by our research team (20, 23, 63–66). This stood true also for HMGB1 levels being higher in overweight/obese PCOS. The positive association of HMGB1 with IGFBP-2 is compatible with the increased inflammatory status in PCOS patients and confirms previous findings in chronic inflammatory diseases characterized by an increase in circulating IGFBP-2 (25, 66).

This study does present some limitations. Indeed, we do not have information on IGFBP proteolysis as this has been shown in a few studies and would further impact IGF bioactivity (67, 68). Furthermore, we are aware that the stimulation protocol for IVF could have had an effect on protein concentrations in FF, but both PCOS and control subjects underwent the same stimulation protocol; thus, the two groups can be compared as in previous studies (20, 43), and for ethical reasons, FF could not have been obtained differently. It should be considered, however, that the study offers a picture of changes in PCOS versus controls at ovulation. The difference in age between PCOS patients and control subjects (mean age difference: 2.7 years) is unlikely to have partially impacted the between-group comparisons because of the physiological decline in circulating and intra-ovarian IGF system components with age. Furthermore, we acknowledge a relatively small number of patients in the overweight/obese control group and cannot entirely exclude that this could have had an effect on the differences between overweight/obese PCOS and overweight/obese controls, especially with regard to IGF-II and IGFBP-4. The means and SD of IGFBP-7 between overweight/obese PCOS and overweight/obese controls are almost identical so it is even less probable that a slight increase in the sample size of the overweight/obese control group could affect the strength of the observation. Moreover, we are aware that tubal factor infertility and unexplained infertility in control subjects may involve alterations in the intrafollicular IGF and inflammatory milieu. However, control subjects were screened to exclude other ovulatory dysfunctions or mild forms of PCOS to avoid any potential confounding factors.

Overall, this study provides an overview of the IGF system in FF obtained from women with PCOS, showing significant changes, revealing and underscoring a role of IGFBP-6 and BP-7, currently poorly studied in PCOS and in humans. In the future, further research should evaluate whether these specific IGFBPs could be used as biomarkers of oocyte quality by evaluating considering also any correlations between IGFBP levels in FF and the rate of pregnancies. The study also highlights reduced IGF-II bioactivity in FF in PCOS. These data provide some useful information on the derangements in PCOS and on possible mechanisms related to comorbidities. Moreover, as the IGF system is related with the development of some cancers (69), somehow the changes described could be possibly related with the increased risk of developing ovarian cancer in these patients (70) that should be addressed by further research.

## Data Availability

The raw data supporting the conclusions of this article will be made available by the authors, without undue reservation.

## References

[1] Lizneva D Suturina L Walker W Brakta S Gavrilova-Jordan L Azziz R . Criteria, prevalence, and phenotypes of polycystic ovary syndrome. Fertil Steril. (2016) 106:6–15. doi: 10.1016/j.fertnstert.2016.05.003. PMID: 27233760

[2] Fauser BC Tarlatzis BC Rebar RW Legro RS Balen AH Lobo R . Consensus on women's health aspects of polycystic ovary syndrome (PCOS): the Amsterdam ESHRE/ASRM-Sponsored 3rd PCOS Consensus Workshop Group. Fertil Steril. (2012) 97:28–38.e25. doi: 10.1016/j.fertnstert.2011.09.024. PMID: 22153789

[3] Joham AE Norman RJ Stener-Victorin E Legro RS Franks S Moran LJ . Polycystic ovary syndrome. Lancet Diabetes Endocrinol. (2022) 10:668–80. doi: 10.1055/s-0041-1735506. PMID: 35934017

[4] Escobar-Morreale HF . Polycystic ovary syndrome: definition, aetiology, diagnosis and treatment. Nat Rev Endocrinol. (2018) 14:270–84. doi: 10.1038/nrendo.2018.24. PMID: 29569621

[5] Barber TM McCarthy MI Wass JA Franks S . Obesity and polycystic ovary syndrome. Clin Endocrinol Oxf. (2006) 65:137–45. doi: 10.1111/cen.14421. PMID: 16886951

[6] Teede HJ Tay CT Laven JJE Dokras A Moran LJ Piltonen TT . Recommendations from the 2023 international evidence-based guideline for the assessment and management of polycystic ovary syndrome. Fertil Steril. (2023) 120:767–93. doi: 10.2139/ssrn.4689131 37589624

[7] Tosi F Bonora E Moghetti P . Insulin resistance in a large cohort of women with polycystic ovary syndrome: a comparison between euglycaemic-hyperinsulinaemic clamp and surrogate indexes. Hum Reprod. (2017) 32:2515–21. doi: 10.1093/humrep/dex308. PMID: 29040529

[8] Liu Y Li J Yan Z Liu D Ma J Tong N . Improvement of insulin sensitivity increases pregnancy rate in infertile PCOS women: a systemic review. Front Endocrinol Lausanne. (2021) 12:657889. doi: 10.3389/fendo.2021.657889. PMID: 33859621 PMC8042389

[9] Mu L Li R Lai Y Zhao Y Qiao J . Adipose insulin resistance is associated with cardiovascular risk factors in polycystic ovary syndrome. J Endocrinol Invest. (2019) 42:541–8. doi: 10.1007/s40618-018-0949-2. PMID: 30206805

[10] Moghetti P Tosi F Castello R Magnani CM Negri C Brun E . The insulin resistance in women with hyperandrogenism is partially reversed by antiandrogen treatment: evidence that androgens impair insulin action in women. J Clin Endocrinol Metab. (1996) 81:952–60. doi: 10.1210/jc.81.3.952. PMID: 8772557

[11] Gilbert EW Tay CT Hiam DS Teede HJ Moran LJ . Comorbidities and complications of polycystic ovary syndrome: an overview of systematic reviews. Clin Endocrinol Oxf. (2018) 89:683–99. doi: 10.1111/cen.13828. PMID: 30099747

[12] Rostamtabar M Esmaeilzadeh S Karkhah A Amiri M Rahmani A Bakouei F . Elevated expression of IL-18 but not IL-1β gene is associated with NALP3 and AIM2 inflammasome in polycystic ovary syndrome. Gene. (2020) 731:144352. doi: 10.1016/j.gene.2020.144352. PMID: 31935500

[13] Sartori C Lazzeroni P Merli S Patianna VD Viaroli F Cirillo F . From placenta to polycystic ovarian syndrome: the role of adipokines. Mediators Inflammation. (2016) 2016:4981916. doi: 10.1155/2016/4981916. PMID: 27746590 PMC5056282

[14] Garruti G Depalo R Vita MG Giampetruzzi F Bellomo Damato A Giorgino F . Adipose tissue, metabolic syndrome and polycystic ovary syndrome: from pathophysiology to treatment. Reprod BioMed Online. (2009) 19:552–63. doi: 10.1016/j.rbmo.2009.05.010. PMID: 19909598

[15] Liu Y Liu H Li Z Fan H Yan X Liu X . The release of peripheral immune inflammatory cytokines promote an inflammatory cascade in PCOS patients via altering the follicular microenvironment. Front Immunol. (2021) 12:685724. doi: 10.3389/fimmu.2021.685724. PMID: 34079559 PMC8165443

[16] González F . Inflammation in polycystic ovary syndrome: underpinning of insulin resistance and ovarian dysfunction. Steroids. (2012) 77:300–5. doi: 10.1016/j.steroids.2011.12.003 PMC3309040 22178787

[17] Amato G Conte M Mazziotti G Lalli E Vitolo G Tucker AT . Serum and follicular fluid cytokines in polycystic ovary syndrome during stimulated cycles. Obstet Gynecol. (2003) 101:1177–82. doi: 10.1016/s0029-7844(03)00233-3. PMID: 12798522

[18] Cho M Kim S Chun S . Relationship between hematologic parameters related to systemic inflammation and insulin resistance-associated metabolic parameters in women with polycystic ovary syndrome. Clin Exp Reprod Med. (2023) 50:206–12. doi: 10.5653/cerm.2023.05932. PMID: 37643835 PMC10477410

[19] González F Rote NS Minium J Kirwan JP . Increased activation of nuclear factor kappaB triggers inflammation and insulin resistance in polycystic ovary syndrome. J Clin Endocrinol Metab. (2006) 91:1508–12. doi: 10.1210/jc.2005-2327 16464947

[20] Cirillo F Catellani C Sartori C Lazzeroni P Morini D Nicoli A . CFTR and FOXO1 gene expression are reduced and high mobility group box 1 (HMGB1) is increased in the ovaries and serum of women with polycystic ovarian syndrome. Gynecol Endocrinol. (2019) 35:842–6. doi: 10.1080/09513590.2019.1599349. PMID: 30964354

[21] Yang H Wang H Chavan SS Andersson U . High mobility group box protein 1 (HMGB1): the prototypical endogenous danger molecule. Mol Med. (2015) 21 Suppl 1:S6–S12. doi: 10.2119/molmed.2015.00087. PMID: 26605648 PMC4661054

[22] Wang Y Zhong J Zhang X Liu Z Yang Y Gong Q . The role of HMGB1 in the pathogenesis of type 2 diabetes. J Diabetes Res. (2016) 2016:2543268. doi: 10.1155/2016/2543268. PMID: 28101517 PMC5215175

[23] Moghetti P Catellani C Sartori C Migazzi M Cirillo F Villani M . Serum HMGB1 levels are independently associated with glucose clamp-derived measures of insulin resistance in women with PCOS. J Endocrinol Invest. (2023) 46:2629–37. doi: 10.1007/s40618-023-02119-y. PMID: 37256493 PMC10632283

[24] Baxter RC . Signaling pathways of the insulin-like growth factor binding proteins. Endocr Rev. (2023) 44:753–78. doi: 10.1210/endrev/bnad008. PMID: 36974712 PMC10502586

[25] Street ME Ziveri MA Spaggiari C Viani I Volta C Grzincich GL . Inflammation is a modulator of the insulin-like growth factor (IGF)/IGF-binding protein system inducing reduced bioactivity of IGFs in cystic fibrosis. Eur J Endocrinol. (2006) 154:47–52. doi: 10.1530/eje.1.02064. PMID: 16381990

[26] Jones JI Clemmons DR . Insulin-like growth factors and their binding proteins: biological actions. Endocr Rev. (1995) 16:3–34. doi: 10.1210/er.16.1.3. PMID: 7758431

[27] Binoux M . The IGF system in metabolism regulation. Diabete Metab. (1995) 21:330–7. 8586149

[28] Katsanos KH Tsatsoulis A Christodoulou D Challa A Katsaraki A Tsianos EV . Reduced serum insulin-like growth factor-1 (IGF-1) and IGF-binding protein-3 levels in adults with inflammatory bowel disease. Growth Hormone IGF Res. (2001) 11:364–7. doi: 10.1054/ghir.2001.0248. PMID: 11914023

[29] Friedrich N Thuesen B Jørgensen T Juul A Spielhagen C Wallaschofksi H . The association between IGF-I and insulin resistance: a general population study in Danish adults. Diabetes Care. (2012) 35:768–73. doi: 10.2337/dc11-1833 PMC3308317 22374641

[30] Savvidis C Kouroglou E Kallistrou E Ragia D Dionysopoulou S Gavriiloglou G . IGFBP-2 in critical illness: a prognostic marker in the growth hormone/insulin-like growth factor axis. Pathophysiology. (2024) 31:621–30. doi: 10.3390/pathophysiology31040045. PMID: 39585162 PMC11587456

[31] Street ME Smerieri A Montanini L Predieri B Iughetti L Valenzise M . Interactions among pro-inflammatory cytokines, IGF system and thyroid function in pre-pubertal obese subjects. J Biol Regul Homeost Agents. (2013) 27:259–66. 23489706

[32] Street ME de'Angelis G Camacho-Hübner C Giovannelli G Ziveri MA Bacchini PL . Relationships between serum IGF-1, IGFBP-2, interleukin-1beta and interleukin-6 in inflammatory bowel disease. Horm Res. (2004) 61:159–64. doi: 10.1159/000075699. PMID: 14691340

[33] Lewitt MS Dent MS Hall K . The insulin-like growth factor system in obesity, insulin resistance and type 2 diabetes mellitus. J Clin Med. (2014) 3:1561–74. doi: 10.3390/jcm3041561. PMID: 26237614 PMC4470198

[34] Kubo H Sawada S Satoh M Asai Y Kodama S Sato T . Insulin-like growth factor-1 levels are associated with high comorbidity of metabolic disorders in obese subjects; a Japanese single-center, retrospective-study. Sci Rep. (2022) 12:20130. doi: 10.1038/s41598-022-23521-1. PMID: 36418379 PMC9684525

[35] Heald AH Kärvestedt L Anderson SG McLaughlin J Knowles A Wong L . Low insulin-like growth factor-II levels predict weight gain in normal weight subjects with type 2 diabetes. Am J Med. (2006) 119:167.e9–167.e15. doi: 10.1016/j.amjmed.2005.08.001. PMID: 16443426

[36] Sandhu MS Gibson JM Heald AH Dunger DB Wareham NJ . Low circulating IGF-II concentrations predict weight gain and obesity in humans. Diabetes. (2003) 52:1403–8. doi: 10.2337/diabetes.52.6.1403. PMID: 12765950

[37] Reinehr T Kleber M Toschke AM Woelfle J Roth CL . Longitudinal association between IGFBP-1 levels and parameters of the metabolic syndrome in obese children before and after weight loss. Int J Pediatr Obes. (2011) 6:236–43. doi: 10.3109/17477166.2010.544739. PMID: 21198359

[38] Mazerbourg S Monget P . Insulin-like growth factor binding proteins and IGFBP proteases: a dynamic system regulating the ovarian folliculogenesis. Front Endocrinol Lausanne. (2018) 9:134. doi: 10.3389/fendo.2018.00134. PMID: 29643837 PMC5890141

[39] Tkachenko OY Wolf S Lawson MS Ting AY Rodrigues JK Xu F . Insulin-like growth factor 2 is produced by antral follicles and promotes preantral follicle development in macaques†. Biol Reprod. (2021) 104:602–10. doi: 10.1093/biolre/ioaa227. PMID: 33348377 PMC7962767

[40] Bøtkjær JA Pors SE Petersen TS Kristensen SG Jeppesen JV Oxvig C . Transcription profile of the insulin-like growth factor signaling pathway during human ovarian follicular development. J Assist Reprod Genet. (2019) 36:889–903. doi: 10.1007/s10815-019-01432-x 30877600 PMC6541695

[41] Wang TH Chang CL Wu HM Chiu YM Chen CK Wang HS . Insulin-like growth factor-II (IGF-II), IGF-binding protein-3 (IGFBP-3), and IGFBP-4 in follicular fluid are associated with oocyte maturation and embryo development. Fertil Steril. (2006) 86:1392–401. doi: 10.1016/j.fertnstert.2006.03.064. PMID: 17070193

[42] Thomas FH Campbell BK Armstrong DG Telfer EE . Effects of IGF-I bioavailability on bovine preantral follicular development *in vitro*. Reproduction. (2007) 133:1121–8. doi: 10.1530/rep-06-0382. PMID: 17636166

[43] Barreca A Del Monte P Ponzani P Artini PG Genazzani AR Minuto F . Intrafollicular insulin-like growth factor-II levels in normally ovulating women and in patients with polycystic ovary syndrome. Fertil Steril. (1996) 65:739–45. doi: 10.1016/s0015-0282(16)58206-5. PMID: 8654631

[44] Thierry van Dessel HJ Lee PD Faessen G Fauser BC Giudice LC . Elevated serum levels of free insulin-like growth factor I in polycystic ovary syndrome. J Clin Endocrinol Metab. (1999) 84:3030–5. doi: 10.1210/jc.84.9.3030. PMID: 10487660

[45] Fowler DJ Nicolaides KH Miell JP . Insulin-like growth factor binding protein-1 (IGFBP-1): a multifunctional role in the human female reproductive tract. Hum Reprod Update. (2000) 6:495–504. doi: 10.1093/humupd/6.5.495. PMID: 11045880

[46] Cataldo NA Giudice LC . Insulin-like growth factor binding protein profiles in human ovarian follicular fluid correlate with follicular functional status. J Clin Endocrinol Metab. (1992) 74:821–9. doi: 10.1210/jc.74.4.821. PMID: 1372322

[47] Schams D Berisha B Kosmann M Einspanier R Amselgruber WM . Possible role of growth hormone, IGFs, and IGF-binding proteins in the regulation of ovarian function in large farm animals. Domest Anim Endocrinol. (1999) 17:279–85. doi: 10.1016/s0739-7240(99)00044-2. PMID: 10527130

[48] Hastie PM Haresign W . Expression of mRNAs encoding insulin-like growth factor (IGF) ligands, IGF receptors and IGF binding proteins during follicular growth and atresia in the ovine ovary throughout the oestrous cycle. Anim Reprod Sci. (2006) 92:284–97. doi: 10.1016/j.anireprosci.2005.05.022. PMID: 16023803

[49] Chen L Hui L Wang Y Yao X Li J . Elevated IGFBP7 expression in follicular granulosa cells promotes PCOS pathogenesis. Biochim Biophys Acta Mol Basis Dis. (2025) 1871:167743. doi: 10.1016/j.bbadis.2025.167743. PMID: 39988179

[50] Przewocki J Łukaszuk A Jakiel G Wocławek-Potocka I Kłosińska K Olszewska J . Proteomic analysis of follicular fluid in polycystic ovary syndrome: insights into protein composition and metabolic pathway alterations. Int J Mol Sci. (2024) 25:11749. doi: 10.3390/ijms252111749. PMID: 39519300 PMC11546118

[51] Rotterdam ESHRE/ASRM-Sponsored PCOS Consensus Workshop Group . Revised 2003 consensus on diagnostic criteria and long-term health risks related to polycystic ovary syndrome. Fertil Steril. (2004) 81:19–25. doi: 10.1093/humrep/deh098. PMID: 14711538

[52] Walenkamp MJ Karperien M Pereira AM Hilhorst-Hofstee Y van Doorn J Chen JW . Homozygous and heterozygous expression of a novel insulin-like growth factor-I mutation. J Clin Endocrinol Metab. (2005) 90:2855–64. doi: 10.1210/jc.2004-1254. PMID: 15769976

[53] Monte APO Barros VRP Santos JM Menezes VG Cavalcante AYP Gouveia BB . Immunohistochemical localization of insulin-like growth factor-1 (IGF-1) in the sheep ovary and the synergistic effect of IGF-1 and FSH on follicular development *in vitro* and LH receptor immunostaining. Theriogenology. (2019) 129:61–9. doi: 10.1016/j.theriogenology.2019.02.005. PMID: 30822644

[54] Rey F Rodríguez FM Salvetti NR Palomar MM Barbeito CG Alfaro NS . Insulin-like growth factor-II and insulin-like growth factor-binding proteins in bovine cystic ovarian disease. J Comp Pathol. (2010) 142:193–204. doi: 10.1016/j.jcpa.2009.11.002. PMID: 19959179

[55] Firmansyah A Chalid MT Farid RB Nusratuddin N . The correlation between insulin-like growth factor binding protein 1 (IGFBP-1) and homeostasis model assessment of insulin resistance (HOMA-IR) in polycystic ovarian syndrome with insulin resistance. Int J Reprod BioMed. (2018) 16:679–82. PMC6350850 30775682

[56] Homburg R Pariente C Lunenfeld B Jacobs HS . The role of insulin-like growth factor-1 (IGF-1) and IGF binding protein-1 (IGFBP-1) in the pathogenesis of polycystic ovary syndrome. Hum Reprod. (1992) 7:1379–83. doi: 10.1093/oxfordjournals.humrep.a137577. PMID: 1283982

[57] Bersinger NA Kollmann Z Von Wolff M . Serum but not follicular fluid cytokine levels are increased in stimulated versus natural cycle IVF: a multiplexed assay study. J Reprod Immunol. (2014) 106:27–33. doi: 10.1016/j.jri.2014.06.003. PMID: 25103590

[58] Hjortebjerg R Kristiansen MR Brandslund I Aa Olsen D Stidsen JV Nielsen JS . Associations between insulin-like growth factor binding protein-2 and insulin sensitivity, metformin, and mortality in persons with T2D. Diabetes Res Clin Pract. (2023) 205:110977. doi: 10.1016/j.diabres.2023.110977. PMID: 37890435

[59] Yang J Zhou W Wu Y Xu L Wang Y Xu Z . Circulating IGFBP-2 levels are inversely associated with the incidence of nonalcoholic fatty liver disease: A cohort study. J Int Med Res. (2020) 48:300060520935219. doi: 10.1177/0300060520935219. PMID: 32762395 PMC7707858

[60] Lee H Oh JY Sung YA . Adipokines, insulin-like growth factor binding protein-3 levels, and insulin sensitivity in women with polycystic ovary syndrome. Korean J Intern Med. (2013) 28:456–63. doi: 10.3904/kjim.2013.28.4.456. PMID: 23864804 PMC3712154

[61] Manousopoulou A Al-Daghri NM Sabico S Garay-Baquero DJ Teng J Alenad A . Polycystic ovary syndrome and insulin physiology: An observational quantitative serum proteomics study in adolescent, normal-weight females. Proteomics Clin Appl. (2019) 13:e1800184. doi: 10.1002/prca.201800184. PMID: 30968585

[62] Amato G Izzo A Tucker AT Bellastella A . Lack of insulin-like growth factor binding protein-3 variation after follicle-stimulating hormone stimulation in women with polycystic ovary syndrome undergoing *in vitro* fertilization. Fertil Steril. (1999) 72:454–7. doi: 10.1016/s0015-0282(99)00288-5. PMID: 10519616

[63] Hu M Zhang Y Lu Y Han J Guo T Cui P . Regulatory mechanisms of HMGB1 and its receptors in polycystic ovary syndrome-driven gravid uterine inflammation. FEBS J. (2023) 290:1874–906. doi: 10.1111/febs.16678. PMID: 36380688 PMC10952262

[64] Li H Zhao X . Predictive value of serum sortilin, HMGB1, and galanin-like peptide for gestational diabetes mellitus in women with polycystic ovary syndrome. Front Endocrinol Lausanne. (2025) 16:1602622. doi: 10.3389/fendo.2025.1602622. PMID: 40458174 PMC12127198

[65] Montanini L Cirillo F Smerieri A Pisi G Giardino I d'Apolito M . HMGB1 is increased by CFTR loss of function, is lowered by insulin, and increases *in vivo* at onset of CFRD. J Clin Endocrinol Metab. (2016) 101:1274–81. doi: 10.1210/jc.2015-3730. PMID: 26760176

[66] Cirillo F Catellani C Lazzeroni P Sartori C Tridenti G Vezzani C . HMGB1 is increased in adolescents with polycystic ovary syndrome (PCOS) and decreases after treatment with myo-inositol (MYO) in combination with alpha-lipoic acid (ALA). Gynecol Endocrinol. (2020) 36:588–93. doi: 10.1080/09513590.2020.1725967. PMID: 32054355

[67] Monget P Mazerbourg S Delpuech T Maurel MC Manière S Zapf J . Pregnancy-associated plasma protein-A is involved in insulin-like growth factor binding protein-2 (IGFBP-2) proteolytic degradation in bovine and porcine preovulatory follicles: identification of cleavage site and characterization of IGFBP-2 degradation. Biol Reprod. (2003) 68:77–86. doi: 10.1095/biolreprod.102.007609. PMID: 12493698

[68] Choi YS Ku SY Jee BC Suh CS Choi YM Kim JG . Comparison of follicular fluid IGF-I, IGF-II, IGFBP-3, IGFBP-4 and PAPP-A concentrations and their ratios between GnRH agonist and GnRH antagonist protocols for controlled ovarian stimulation in IVF-embryo transfer patients. Hum Reprod. (2006) 21:2015–21. doi: 10.1093/humrep/del091 16601008

[69] Weroha SJ Haluska P . The insulin-like growth factor system in cancer. Endocrinol Metab Clin North Am. (2012) 41:335–vi. doi: 10.1016/j.ecl.2012.04.014 22682634 PMC3614012

[70] Kurbaniyazova S Nurkhasimova R Ayazbekov A Khudaibergenova S Kulbayeva S Mirzakhmetova D . Systematic review of the literature: estimation of the most common gynecological disorders and associated factors among Kazakhstani adolescents. Future Sci OA. (2026) 12:2599726. doi: 10.1080/20565623.2025.2599726. PMID: 41388843 PMC12710883

