# Supplementary material for: IGF-II, IGFBP-4, -6, and -7, and HMGB1 show changes in follicular fluid in PCOS
Source: Front Endocrinol (Lausanne). 2026 Jun 5;17:1856759. doi: 10.3389/fendo.2026.1856759 (PMC13278883; doi:10.3389/fendo.2026.1856759)
Supplement: Supplementary file 1 [file DataSheet1.docx]

Supplementary Material

# Supplementary Tables

**Supplementary Table 1**. Main clinical and biochemical characteristics of PCOS and control women.

| **Parameter** | **PCOS patients** | **control subjects** |
| --- | --- | --- |
|  | n=70 | n=70 |
| **Chronological Age (yr, mean±SD)** | 34.1±4.7 | 36.8± 3.8 |
| **BMI (kg/m^2^, mean±SD [range])** | 25.6±5.6 [17.6-39.3] | 24.0±5.0 [17.2-38.7] |
| **Hirsute (n)** | 16 | 0 |
| **Amenorrhoic (n)** | 6 | 0 |
| **Oligomenorrhoic (n)** | 31 | 0 |
| **Regularly cycling (n)** | 33 | 70 |
| **Any features of PCOS at ultrasonography** | 70 | 0 |
| **E2 (pg/ml, mean±SD)** | 1860±1285 | 1389±889 |

Abbreviations: yr = years, n = number, BMI= body mass index, E2= estradiol at the time of oocyte retrieval, SD=standard deviation

**Supplementary Table 2**. Technical characteristics of the assays.

| **Assay** | **Intra-assay CV** | **Inter-assay CV** | **Upper limit of detection** | **Lower limit of detection** |
| --- | --- | --- | --- | --- |
| **IGF-I** | <5% | <6.5% | 5.2 ng/ml | 0.1 ng/ml |
| **IGF-II** | <3.5% | <9.5% | 780.4 pg/ml | 12.5 pg/ml |
| **IGFBP-1** | <10% | <12% | 1686.4 pg/ml | 2.7 pg/ml |
| **IGFBP-2** | <10% | <10% | 80 ng/ml | 2.1 ng/ml |
| **IGFBP-3** | <5.5% | <8.5% | 50.3 ng/ml | 0.8 ng/ml |
| **IGFBP-4** | <10% | <12% | 99.9 ng/ml | 0.8 ng/ml |
| **IGFBP-5** | <10% | <12% | 804.8 ng/ml | 8 ng/ml |
| **IGFBP-6** | <10% | <12% | 55461 pg/ml | 27 pg/ml |
| **IGFBP-7** | <10% | <12% | 35 ng/ml | 0.03 ng/ml |
| **HMGB1** | <13.7% | <13.7% | 80 ng/ml | 2.2 ng/ml |

# Supplementary Figures


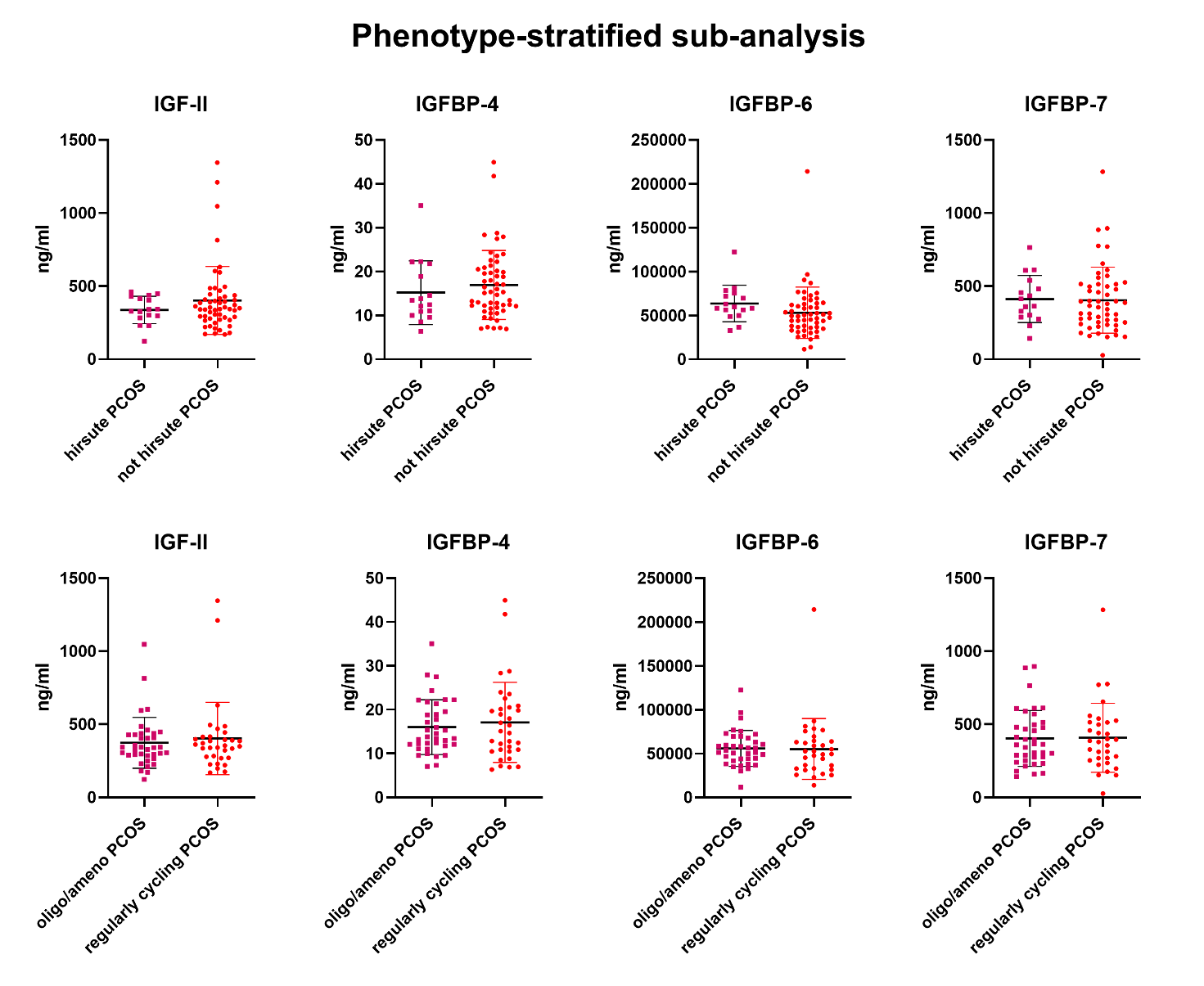


**Supplementary Figure 1.** **Effect of the different PCOS phenotypes on IGF-II, IGFBP-4, IGFBP-6, IGFBP-7.** The following comparisons have been performed: hirsute (n = 16) vs not hirsute (n= 54); amenorrhoeic/oligomenorrhoeic (n = 37) vs regularly cycling (n = 33) women. p<0.05 were considered significant.


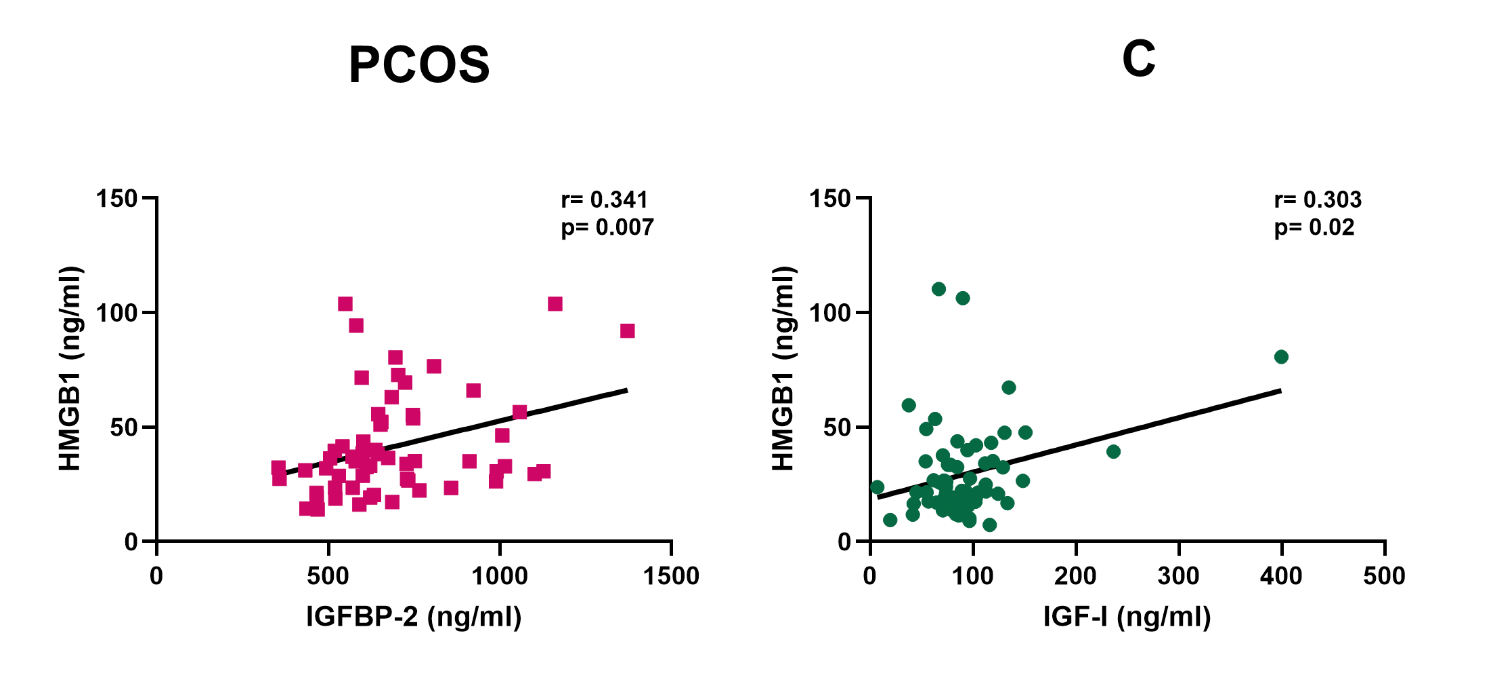


**Supplementary Figure 2.** **Correlations among HMGB1 and IGF system proteins in FF of women with PCOS and controls.** PCOS n=61; controls (C) n=64. Only correlations which reached the statistical significance set to p<0.05 and with an R > 0.30 are shown.
